# Association of gait, balance, and fall risk with dementia in Down syndrome: a systematic review of the literature

**DOI:** 10.1590/1980-5764-DN-2025-0442

**Published:** 2026-07-20

**Authors:** Aline de Souza Gonçalves Gomes da Conceição, Luciana Mascarenhas Fonseca, Orestes Vicente Forlenza

**Affiliations:** 1 Universidade de São Paulo, Faculdade de Medicina, Departamento e Instituto de Psiquiatria, Laboratório de Neurociência (LIM-27), São Paulo SP, Brazil.; 2 The Krieger Klein Alzheimer's Research Center, Rutgers Health, NJ, United States.; 3 Universidade de São Paulo, Faculdade de Medicina, Departamento e Instituto de Psiquiatria, Grupo de Pesquisa em Idosos, São Paulo SP, Brazil.

**Keywords:** Trisomy 21, Intellectual Disability, Dementia, Gait, Postural Balance, Trissomia do Cromossomo 21, Deficiência Intelectual, Demência, Marcha, Equilíbrio Postural

## Abstract

Advances in healthcare have led to increased life expectancy among individuals with Down syndrome, resulting in a growing population of older adults within this group. As a result, the prevalence of cognitive decline and dementia has increased, largely due to the genetic predisposition of Down syndrome to early and significant accumulation of brain amyloid and intensified formation of neurofibrillary tangles. Early detection of functional alterations, particularly those related to gait and balance, may serve as a valuable clinical marker of emerging neurocognitive impairment.

**Objective:**

To synthesize the current evidence on the relationship between gait, cognitive decline, and dementia in adults and older adults with Down syndrome.

**Methods:**

A systematic search was conducted in the PubMed database in accordance with the Preferred Reporting Items for Systematic Reviews and Meta-Analyses (PRISMA) 2020 guidelines. Original observational studies evaluating gait, balance, or fall risk in relation to cognitive decline or dementia in adults with Down syndrome were included. Methodological quality was assessed using the Joanna Briggs Institute critical appraisal tools.

**Results:**

Thirty-seven records were identified, of which five original studies met the inclusion criteria. Overall, the findings suggest an association between gait alterations, balance, and cognitive decline in individuals with Down syndrome. Commonly reported abnormalities included reduced gait speed, increased gait variability, inconsistency in gait parameters, and impaired balance. However, heterogeneity in study design, different measurement protocols, and small sample sizes limited the comparability and generalizability of the results.

**Conclusion:**

Gait assessment holds promise as a non-invasive, accessible approach for early identification of cognitive decline in individuals with Down syndrome. Future research should prioritize longitudinal designs, standardized assessment protocols, and larger representative samples to inform targeted and evidence-based clinical interventions.

## RESUMO

Avanços na área da saúde levaram ao aumento da expectativa de vida de indivíduos com síndrome de Down (SD), resultando em uma população crescente de adultos e idosos nesse grupo. Como consequência, houve aumento da prevalência de declínio cognitivo e demência, em grande parte em razão da predisposição genética da SD para acúmulo precoce e significativo de beta-amiloide cerebral e intensificação da formação de emaranhados neurofibrilares. A detecção precoce de alterações funcionais, particularmente aquelas relacionadas à marcha e ao equilíbrio, pode constituir um marcador clínico relevante de comprometimento neurocognitivo emergente.

**Objetivo:**

Sintetizar as evidências atuais sobre a relação entre marcha, declínio cognitivo e demência em adultos e idosos com SD.

**Métodos:**

Foi realizada uma busca sistemática na base de dados United States National Library of Medicine (PubMed), de acordo com as diretrizes do Preferred Reporting Items for Systematic Reviews and Meta-Analyses (PRISMA) 2020. Foram incluídos estudos observacionais originais que avaliaram marcha, equilíbrio ou risco de quedas em relação ao declínio cognitivo ou demência em adultos com SD. A qualidade metodológica foi avaliada por meio das ferramentas de avaliação crítica do Joanna Briggs Institute.

**Resultados:**

Foram identificados 37 registros, dos quais cinco estudos originais atenderam aos critérios de inclusão. De modo geral, os achados sugerem associação entre alterações na marcha, no equilíbrio e declínio cognitivo em indivíduos com SD. As alterações mais frequentemente relatadas incluíram redução da velocidade da marcha, aumento da variabilidade desta, inconsistência nos parâmetros de marcha e prejuízo do equilíbrio. Contudo, a heterogeneidade no delineamento dos estudos, nos protocolos de mensuração e o tamanho reduzido das amostras limitam a comparabilidade e a generalização dos resultados.

**Conclusão:**

A avaliação da marcha apresenta potencial como abordagem não invasiva e acessível para a identificação precoce de declínio cognitivo em indivíduos com SD. Estudos futuros devem priorizar delineamentos longitudinais, protocolos padronizados de avaliação e amostras maiores e representativas, a fim de subsidiar intervenções clínicas direcionadas e baseadas em evidências.

## INTRODUCTION

People with Down syndrome (DS) have trisomy of chromosome 21, resulting in three copies of all the genes on this chromosome, including the APP gene (amyloid precursor protein)^1-3^. Increased APP gene dosage leads to overproduction of the precursor protein, which is subsequently cleaved to form the beta-amyloid (Aβ) peptide, the main component of the senile plaques characteristic of Alzheimer's disease (AD). This mechanism explains the high prevalence and early onset of AD in individuals with DS, who invariably develop the typical neuropathology of the disease over the course of their lives^1,2,4^. DS is currently considered, according to medical literature consensus, a segmental progeroid syndrome. Multiple studies have shown that individuals with DS exhibit accelerated biological aging, with early manifestations of age-related conditions such as cognitive decline, neurodegenerative diseases (including early-onset AD), immunosenescence, osteoporosis, cardiovascular alterations, and a higher prevalence of geriatric comorbidities at a young age^1,4,5^. Overexpression of the APP gene in chromosome 21 in DS is associated with increased amyloid levels in the brain and amyloid plaques in all individuals with DS over the age of 40, and a 90% lifetime risk of developing dementia^3^. This pathological process follows a pattern similar to that of autosomal dominant AD, with deposition of tau neurofibrillary tangles (NFT) occurring in the 40s and 50s, followed by progressive neurodegeneration^6,7^. DS is the most common genetic cause of intellectual disability (ID), with most individuals with DS classified as having mild to moderate ID. Their cognitive profile demonstrates strengths in visual learning but weaknesses in expressive language, verbal working memory, and episodic memory^8^. Because many adults with DS have limited verbal skills, traditional cognitive assessments may be particularly challenging. Therefore, identifying functional markers, such as gait alterations, may contribute to early screening strategies^9^. Evidence on the experiences and daily functional performance of adults with DS while aging is limited^10,11^. Cognitive alterations often result in impaired functional abilities, including basic self-care activities, such as eating and bathing^12^. These changes in functional activity and performance have significant implications for disease staging in dementia-related disorders. Falls and functional mobility, for example, are associated with clinical measures of cognition and may serve as non-cognitive behavioral markers of AD^11^.

In the general population, a decline in gait performance is associated with the development of AD and may precede cognitive impairment^13^. Recent evidence indicates that gait dysfunction already occurs in prodromal stages, such as in Mild Cognitive Impairment, characterized by deficits in speed, symmetry, and motor execution under dual task^13,14^.

Although it is clear that mobility is associated with attention and executive functions, it has also been suggested that memory and other cognitive abilities, such as praxis, influence changes in mobility, especially gait speed^9^. Several brain regions are known to be involved in specific cognitive functions related to gait. Neuroimaging studies have identified cortical and subcortical areas that play crucial roles in gait control.

Key neural regions are implicated in the integration of motor and cognitive processes underlying gait and balance. Among the principal regions, the prefrontal cortex is responsible for motor planning, attentional control, and executive functions^15,16^, whereas the parietal cortex mediates sensory integration and spatial perception^17^.

The primary motor cortex initiates and regulates voluntary movement, interacting with the basal ganglia to ensure automatization of gait and fine motor control^18,19^. The cerebellum contributes to coordination, postural adjustments, and balance refinement^20,21^, while the brainstem hosts locomotor centers and reflex pathways essential for basic postural and motor integration^21^. The corpus callosum enables inter-hemispheric communication required for bilateral coordination^20,22^, and the hippocampus supports spatial memory, navigation, and orientation^23^. Collectively, these interconnected regions form a distributed neural network, the dysfunction of which has been consistently associated with gait disturbances and increased fall risk in neurodegenerative conditions, including AD in DS^13,21,24^.

Given the well-established associations between gait disturbances, increased fall risk, and dementia in the general population^21^, it is critical to examine these factors in individuals with DS, a group already at elevated risk for early-onset AD^25^. While a growing body of research supports the idea that physical and functional changes may serve as early indicators of cognitive decline, the literature specifically addressing how these changes manifest in aging adults with DS remains limited^9,10,12,13^. A comprehensive literature review is therefore essential to synthesize the current knowledge, identify gaps, and clarify whether gait abnormalities and fall risk could serve as early, non-invasive markers of dementia progression in DS. Such insights could have significant implications for early intervention and the development of targeted strategies to support aging in this unique population. The current systematic review aims to synthesize the scientific evidence on changes in gait, balance, and risk of falls associated with cognitive decline in individuals with DS. To this end, relevant studies published in journals indexed in the PubMed database were identified and analyzed.

## METHODS

This systematic review was conducted and reported in accordance with the Preferred Reporting Items for Systematic Reviews and Meta-Analyses (PRISMA) 2020 statement^26^ and the Joanna Briggs Institute (JBI) Manual for Evidence Synthesis^27^. The review question was structured using the PICO framework, in which the population (P) comprised adults and older adults with DS; the exposure of interest (I) included gait, balance, and fall-related parameters; comparators (C), when available, included individuals without dementia or with different levels of gait performance; and outcomes (O) included cognitive decline, dementia, and AD.

### Search strategy

A systematic literature search was performed in the PubMed database using predefined keywords and Boolean operators: ("Down syndrome" AND dementia) AND (gait OR balance OR "fall risk"). Eligible studies were published between 1995 and 2025 and available in English, Spanish, or Portuguese. All retrieved records were imported into EndNote for reference management and identification of duplicate records.

This search strategy was relatively restricted, as it relied on a single database and a limited set of keywords, which may have omitted relevant studies indexed elsewhere or using alternative terminology.

### Eligibility criteria

Eligibility criteria were defined a priori. We included original observational studies, cross-sectional or longitudinal, involving adults or older adults with DS that assessed gait, balance, and/or fall risk and examined their association with cognitive decline or dementia. Studies exclusively involving children or adolescents were excluded. Narrative reviews, systematic reviews, editorials, and opinion articles were excluded from the main analysis.

### Study selection

Study selection was conducted in two sequential stages:

- screening of titles and abstracts and
- full-text assessment for eligibility.

Two reviewers independently screened all records according to the predefined inclusion and exclusion criteria. Disagreements at any stage were resolved by consensus. The reference management software EndNote was used to organize records and support the screening process. The complete study selection process is summarized in the PRISMA 2020 flow diagram.

### Methodological quality assessment and data synthesis

Methodological quality and risk of bias were independently assessed by two reviewers using JBI critical appraisal tools, selected according to study design. Disagreements were resolved by consensus. Studies that failed to meet minimum methodological adequacy were excluded during full-text assessment. Owing to substantial heterogeneity across studies in terms of design, assessment protocols, and outcome measures, findings were synthesized using a narrative approach, focusing on patterns of association between gait-related measures, balance, fall risk, and cognitive outcomes in adults with DS. Methodological quality was assessed using the standardized JBI Critical Appraisal Checklists, selected according to study design. Cross-sectional studies were evaluated using the JBI Critical Appraisal Checklist for Analytical Cross-Sectional Studies, whereas longitudinal cohort studies were assessed using the JBI Critical Appraisal Checklist for Cohort Studies (Supplementary Material Table S1). Two reviewers independently appraised each study. Each checklist item was rated as "yes," "no," "unclear," or "not applicable." Disagreements were resolved through discussion and consensus. The involvement of a third reviewer was not required^28^.

The results of the methodological quality assessment for the included studies are shown in Table S1.

## RESULTS

The initial search of electronic databases identified 37 records in PubMed. Eighteen records were excluded after full-text assessment because they did not address the research question or failed to meet minimum methodological adequacy criteria based on JBI critical appraisal. Specifically, ten studies did not meet the population eligibility criteria, and eight did not meet methodological quality requirements. No duplicate records were identified

Overall, the studies included demonstrated moderate to high methodological quality (Table S1).

The database search identified 37 records in PubMed. After title and abstract screening, records that did not meet the predefined inclusion criteria were excluded, including review articles and studies not addressing the target population. Full-text assessment was conducted for 19 articles, of which 14 were excluded due to lack of relevance to adults with DS or failure to meet minimum methodological adequacy criteria based on JBI critical appraisal. Ultimately, five original studies fulfilled all eligibility criteria and were included in the systematic review. The complete study selection process is summarized in the PRISMA 2020 flow diagram.

**Figure 1:**
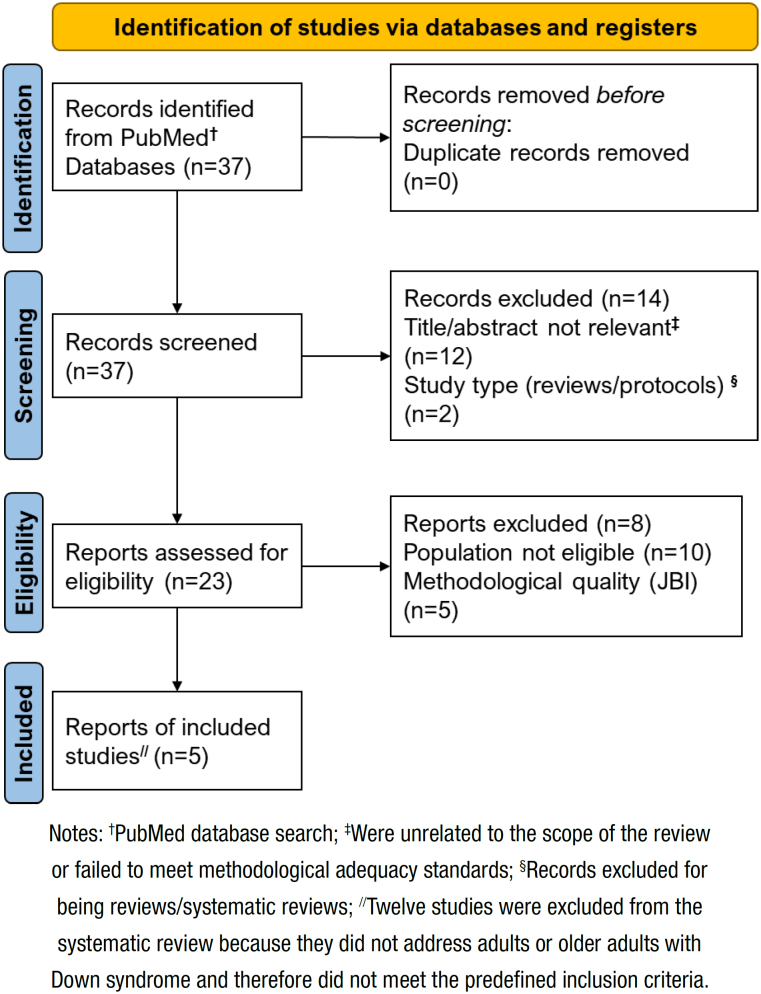
PRISMA 2020 flow diagram for new systematic reviews, which included searches of databases and registers only.

Source: Page et al.^26^

Although we included studies available in English, Spanish, and Portuguese in the search, we only found studies in English. A diagram illustrating the selection process is shown in Figure 1.

Two studies^12,29^ were Reviews and Systematic Reviews and are briefly discussed but not included in Table S1. The studies included in the analysis varied in terms of their objectives, methods, and types. Among the original research articles, five specifically addressed gait disturbances as potential predictors of cognitive decline in adults with DS^9,10,13,30,31^, and are profiled in Table 1 ^9-11,13,30^.

**Table 1:** Characteristics of studies examining gait, balance, fall risk, and cognitive outcomes in adults with Down syndrome.

Author	Country	Study design	Sample (n; age)	Gait/balance/fall assessment	Cognitive or dementia-related outcomes	Main findings relevant to the review question
Conceição et al.^9^	Brazil	Cross-sectional	n=66; 20–64 years	Performance-Oriented Mobility Assessment (POMA); Body Mass Index	CAMCOG-DS; clinical classification (stable cognition, prodromal dementia, dementia)	Poorer gait and balance performance were associated with prodromal dementia and dementia, supporting gait impairment as a functional marker of cognitive decline in adults with Down syndrome.
Van Pelt et al.^10^	USA	Cross-sectional	n=29; 25–59 years	Instrumented gait analysis under single- and dual-task conditions (GAITRite®)	Brief Praxis Test; Severe Impairment Battery; clinical consensus diagnosis	Dual-task gait paradigms revealed age-related slowing and associations with cognitive performance, suggesting cognitive–motor interference as an early marker of cognitive vulnerability.
Barry et al.^13^	USA	Longitudinal cohort (32 months)	n=218; 25–72 years	Tinetti Gait; motion capture and pressure-sensitive walkways	DSMSE; mCRT; NTG-EDSD; consensus diagnosis (stable, MCI-DS, dementia)	Baseline gait impairments and longitudinal gait decline were associated with incident dementia, supporting gait measures as prodromal indicators of Alzheimer's disease in adults with Down syndrome.
Leach et al.^30^	USA	Longitudinal cohort	n=22; mean 37 ± 7.5 years	GAITRite® (step time variability); DTI–MRI	Vineland Adaptive Behavior Scales; Brief Praxis Test; Severe Impairment Battery	Increased gait variability was associated with reduced white matter integrity and poorer adaptive functioning, indicating early neurobiological changes related to dementia risk.
Washington et al.^11^	USA	Cross-sectional	n=43; mean 30 ± 12 years	Timed Up and Go; Sit-to-Stand; grip strength	DSQIID; Waisman Activities of Daily Living Scale	Worse physical function and slower gait were associated with higher dementia risk symptoms and reduced independence, highlighting functional mobility as an early indicator of dementia risk.

Abbreviations: CAMCOG-DS, Cambridge Cognitive Examination-Down Syndrome; DSMSE, Down Syndrome Mental Status Examination; mCRT, modified Cued Recall Test; NTG-EDSD, National Task Group – Early Detection Screen for Dementia; MCI-DS, Mild Cognitive Impairment in Down Syndrome; DTI-MRI, Diffusion Tensor Imaging – Magnetic Resonance Imaging; DSQIID, Dementia Screening Questionnaire for Individuals with Intellectual Disabilities.

### Original research articles

Five original studies examined the association between gait, balance, fall risk, and cognitive outcomes in adults with DS, including three cross-sectional investigations and two longitudinal cohort studies (Table S1)^9-11,13,30^. All studies assessed objective measures of gait or functional mobility, while cognitive or dementia-related outcomes were evaluated using standardized neuropsychological tests, informant-based questionnaires, or multidisciplinary consensus diagnoses. Across studies, outcomes related to cognition or dementia included global cognitive performance, dementia risk symptoms, incident dementia, or functional decline associated with neurodegenerative processes.

Overall, the direction of associations was highly consistent across studies. Poorer gait, balance, or functional mobility — manifested as reduced gait speed, increased gait variability, impaired balance, or worse performance on functional mobility tests — was systematically associated with worse cognitive performance, higher dementia risk, or progression to dementia. Cross-sectional studies consistently reported concurrent associations between mobility impairment and cognitive vulnerability, while longitudinal studies provided convergent evidence that baseline gait abnormalities and subsequent gait decline were associated with incident dementia, rather than with earlier mild cognitive impairment stages. Convergences across studies support gait and functional mobility as clinically relevant markers of dementia risk in adults with DS. Minor inconsistencies were observed regarding the ability of gait measures to discriminate early cognitive impairment stages, likely reflecting differences in study design, sample size, and assessment protocols rather than conflicting evidence.

### Methods used for evaluating gait and balance in Down syndrome

Across the studies included, gait, balance, and functional mobility were assessed using a limited set of standardized clinical and instrumented tools. Gait and balance were most frequently evaluated using the Performance-Oriented Mobility Assessment (POMA/Tinetti)^32^, instrumented gait analysis systems, such as GAITRite™^33^, and clinical functional mobility tests, including the Timed Up and Go (TUG)^34^ and Sit-to-Stand (STS)^33^. Some studies also incorporated quantitative motion capture or pressure-sensitive walkways, while others combined clinical mobility tests with measures of muscle strength or functional performance. Overall, these instruments were selected to capture spatiotemporal gait parameters, balance performance, and functional mobility relevant to fall risk and daily functioning. Details of the specific instruments used in each study are summarized in Table 2.

**Table 2:** Instruments used to assess gait, balance, and functional mobility.

Instrument/Name	Domain assessed	Description	Relevant findings/cut-offs	Specific for DS or ID
POMA – Performance-Oriented Mobility Assessment	Balance and gait	Clinical tool with two subscales (Balance: 9 items; Gait: 7 items); total score 28 points.	Scores <19 indicate high fall risk; applied in Down syndrome, showing an association with prodromal dementia.	No
TUG – Timed Up and Go	Mobility/Function	Functional mobility test: stand up, walk 3 meters, turn, sit. Timed in seconds.	>12s indicates impaired mobility/fall risk; in Down syndrome, associated with dementia risk symptoms.	No
STS – Sit-to-Stand Test	Lower limb strength	Measures the number of chair rises (usually 5x) or time to complete; an indicator of frailty.	Slower times are linked to frailty and dementia risk in Down syndrome.	Yes
GAITRite® System	Quantitative gait	Instrumented walkway; records spatiotemporal gait parameters.	Sensitive to dual-task effects and early Alzheimer's disease changes; feasible in Down syndrome.	No
Motion capture/pressure-sensitive walkways	Gait	Measures velocity, stride length, and variability using cameras or pressure mats.	Identified gait alterations predictive of Alzheimer's disease in aging Down syndrome.	No

Abbreviations: DS, Down syndrome; ID, Intellectual Disability.

### Instruments used to assess cognitive decline and dementia in Down syndrome

Cognitive decline and dementia-related outcomes were evaluated using a combination of standardized neuropsychological instruments, informant-based questionnaires, and multidisciplinary consensus diagnoses. Commonly used tools included the Cambridge Examination for Mental Disorders of Older People with Down's Syndrome (CAMDEX-DS)^35^, the Down Syndrome Mental Status Examination (DSMSE)^36^, and caregiver-reported screening instruments such as the Dementia Screening Questionnaire for Individuals with Intellectual Disabilities (DSQIID)^37^. In longitudinal studies, dementia status was typically established through multidisciplinary consensus^9^ integrating cognitive testing, functional assessments, and clinical information. Overall, these approaches allowed for the identification of cognitive impairment, dementia risk, and incident dementia using methods adapted to the intellectual and functional profiles of adults with DS. A summary of cognitive assessment tools and diagnostic criteria is provided in Table S1.

## DISCUSSION

In this systematic review, gait emerged as a promising functional marker for the early identification of cognitive decline and dementia risk in individuals with DS. However, the available evidence remains limited. Among the studies included, the strength of the observed associations should be interpreted considering the methodological quality of the studies included, which ranged from moderate to high, with stronger evidence derived from longitudinal designs (Table 2). Notably, only five original investigations specifically examined objective measures of gait, balance, or fall risk in adults with DS in association with cognitive impairment or dementia. Despite this limited number, the findings consistently suggest that alterations in gait performance are associated with cognitive decline and progression to dementia in this population^9-11,13,30^.

Across the studies included, commonly reported gait abnormalities included reduced gait speed, increased gait variability, impaired balance, and decreased functional mobility. These alterations mirror findings from the general population, in which gait decline has been shown to precede or accompany cognitive impairment and AD^13,14^. Importantly, gait assessment represents a low-cost, non-invasive, and clinically feasible approach, even in individuals with intellectual disability, making it a particularly attractive screening tool in the DS population.

Longitudinal evidence further supports the relevance of gait as an early marker of neurodegeneration. Barry et al.^13^ demonstrated that baseline gait impairments, particularly reduced gait speed and increased variability, were associated with incident dementia over follow-up, suggesting that mobility decline may emerge near the transition from cognitive stability to dementia in DS. These findings align with neuroimaging evidence linking gait disturbances to amyloid and tau pathology, hippocampal atrophy, and white matter disruption in AD^13,14,21^. Together, these results reinforce the biological plausibility of gait alterations as a behavioral manifestation of underlying neurodegenerative processes.

Other studies highlighted the relevance of functional mobility and fall risk. Washington et al.^11^ reported significant associations between poorer performance on functional mobility tests, increased fall risk, and higher dementia risk scores, emphasizing the clinical relevance of integrating mobility assessments into routine evaluations. Leach et al.^30^ further demonstrated that increased gait variability was associated with reduced white matter integrity and poorer adaptive functioning, supporting gait variability as a sensitive marker of early neural impairment. Dual-task paradigms, explored by Van Pelt et al.^10^, also showed promise for detecting subtle cognitive-motor interference not captured by single-task gait assessments, underscoring the contribution of executive dysfunction to mobility decline.

Although the primary focus of this review was the systematic synthesis of original empirical studies, the limited number of investigations available supports the relevance of incorporating contextual evidence from reviews and conceptual articles. In this regard, Anderson-Mooney et al.^12^ conducted a systematic review with a predominantly conceptual orientation, emphasizing the Motoric Cognitive Risk (MCR) syndrome framework and proposing gait disturbances as potential prodromal indicators of dementia in DS. While this perspective is valuable for advancing theoretical understanding, the review did not apply the same level of methodological rigor as the present study, particularly regarding predefined eligibility criteria, standardized assessment of gait, and differentiation between cross-sectional and longitudinal evidence.

Similarly, Covelli et al.^29^ provided a systematic review of ageing in individuals with DS from a biopsychosocial perspective, based on the International Classification of Functioning, Disability and Health framework. Their findings highlighted that the literature remains largely focused on medical and physical impairments, with limited integration of environmental and contextual factors influencing functional outcomes. Notably, environmental determinants were rarely addressed, underscoring an important gap in the field. Together, these reviews help contextualize the present findings, identifying gaps in the literature and reinforcing the need for more comprehensive and multidimensional approaches to the study of ageing, mobility, and dementia in DS.

Despite the relevance of these findings, this review has important methodological limitations that must be acknowledged. First, the literature search was restricted to a single database (PubMed), which may have resulted in location bias and omitted relevant studies indexed exclusively in other databases, such as Embase, PsycINFO, Scopus, or CINAHL. Consequently, the small number of studies included should not be interpreted as definitive evidence of limited research activity in this field. In addition, this review lacked a previously registered protocol, which may increase the risk of selective reporting.

The studies included also exhibited substantial methodological heterogeneity, including differences in study design (cross-sectional versus longitudinal), gait and balance assessment tools, cognitive and functional measures, and diagnostic criteria for dementia. Sample sizes were generally small, age ranges varied widely, and not all instruments were specifically adapted for individuals with DS or intellectual disability. These factors limited direct comparison across studies, precluded quantitative synthesis, and reduced the generalizability of the findings.

The small number of original studies included limits the strength of the conclusions and reflects the scarcity of empirical research addressing gait, balance, fall risk, and dementia-related outcomes in adults with DS. Potential publication bias cannot be excluded, as studies with null findings may be underrepresented. Therefore, the observed associations should be interpreted as suggestive rather than definitive, highlighting the need for future multicenter longitudinal studies, broader search strategies across multiple databases, protocol registration, and standardized, DS-adapted assessment approaches.

### Future studies

Future studies should adopt longitudinal, multicenter designs, apply standardized and DS-adapted gait and cognitive assessment tools, and integrate functional measures with neuroimaging and biomarker data. Such approaches are essential for determining the temporal relationship between mobility decline and cognitive deterioration and for establishing the validity of gait assessment as a reliable tool for early detection and monitoring of dementia in individuals with DS.

## Data Availability

The datasets generated and/or analyzed during the current study are available from the corresponding author upon reasonable request.
