# Supplementary material for: Association of gait, balance, and fall risk with dementia in Down syndrome: a systematic review of the literature
Source: Dement Neuropsychol. 2026 Jul 20;20:e20250442. doi: 10.1590/1980-5764-DN-2025-0442 (PMC13387804; doi:10.1590/1980-5764-DN-2025-0442)
Supplement: Supplementary Material [file 1980-5764-dn-20-e20250442-Suppl01.docx]

**Supplementary Material**

**Table S1. Methodological quality assessment of included studies using Joanna Briggs Institute (JBI) Critical Appraisal Checklists**

Rating categories: Yes / No / Unclear / Not applicable (N/A). Overall appraisal reflects a qualitative judgment based on domain-level appraisal (no numerical scoring).

**A. Cross-sectional studies – JBI Critical Appraisal Checklist for Analytical Cross-Sectional Studies**

| **Study** | **Ref.** | **Design** | **Q1** | **Q2** | **Q3** | **Q4** | **Q5** | **Q6** | **Q7** | **Q8** | **Overall appraisal** |
| --- | --- | --- | --- | --- | --- | --- | --- | --- | --- | --- | --- |
| Conceição et al., 2023 | 9 | Cross-sectional | Yes | Yes | Yes | Yes | Yes | Yes | Yes | Unclear | Moderate–High |
| Van Pelt et al., 2020 | 10 | Cross-sectional | Yes | Yes | Yes | Yes | Unclear | Yes | Unclear | Unclear | Moderate |
| Washington et al., 2024 | 27 | Cross-sectional | Yes | Yes | Yes | Yes | Yes | Yes | Yes | Yes | High |

JBI domains (Analytical Cross-Sectional Studies): Q1 Inclusion criteria clearly defined; Q2 Study subjects and setting described; Q3 Exposure measured validly and reliably; Q4 Objective, standard criteria used for condition measurement; Q5 Confounding factors identified; Q6 Strategies to deal with confounding stated; Q7 Outcomes measured validly and reliably; Q8 Appropriate statistical analysis.

**B. Longitudinal cohort studies – JBI Critical Appraisal Checklist for Cohort Studies**

| **Study** | **Ref.** | **Design** | **Q1** | **Q2** | **Q3** | **Q4** | **Q5** | **Q6** | **Q7** | **Q8** | **Q9** | **Q10** | **Q11** | **Overall appraisal** |
| --- | --- | --- | --- | --- | --- | --- | --- | --- | --- | --- | --- | --- | --- | --- |
| Barry et al., 2025 | 13 | Cohort (longitudinal) | Yes | Yes | Yes | Yes | Yes | Yes | Yes | Yes | Yes | Yes | Yes | High |
| Leach et al., 2025 | 26 | Cohort (longitudinal) | Yes | Yes | Yes | Yes | Unclear | Yes | Yes | Yes | Unclear | Yes | Yes | Moderate–High |

JBI domains (Cohort Studies): Q1 Groups similar and recruited from the same population; Q2 Exposures measured similarly; Q3 Exposure measured validly and reliably; Q4 Confounding factors identified; Q5 Strategies to deal with confounding stated; Q6 Participants free of outcome at baseline; Q7 Outcomes measured validly and reliably; Q8 Follow-up time reported and sufficient; Q9 Follow-up complete or adequately addressed; Q10 Strategies to address incomplete follow-up; Q11 Appropriate statistical analysis.

Methodological note: Overall methodological quality ranged from moderate to high. Main limitations identified across studies included small sample sizes, incomplete adjustment for confounders, and limited reporting of follow-up information in some investigations. Longitudinal cohort studies provided stronger evidence for associations between gait alterations and dementia risk.
